# Interfacial adaptation of newly prepared nano-tricalcium silicate-58s bioactive glass-based endodontic sealer

**DOI:** 10.34172/joddd.40729

**Published:** 2024-06-24

**Authors:** Nawal A. Al-Sabawi, Sawsan Hameed Al-Jubori

**Affiliations:** Department of Conservative Dentistry, College of Dentistry, University of Mosul, Mosul, Iraq

**Keywords:** Bioactive glass, C3S-BG-P, Endodontic sealer, Interfacial adaptation, Marginal gaps

## Abstract

**Background.:**

The sealer’s interfacial adaptability is one of the critical factors for successful root canal therapy. This study evaluated and compared the interfacial adaptability of newly prepared nano-tricalcium silicate-58s bioactive glass-based endodontic sealer (C_3_ S-BG-P) to root dentin with two bioactive sealers Nishika Canal Sealer BG and BioRoot^TM^ RCS.

**Methods.:**

Thirty newly extracted single-rooted lower premolars were decoronated and instrumented. The roots were assigned to three groups: C_3_ S-BG-P, Nishika Canal Sealer BG, and BioRoot^TM^ RCS (n=10) and obturated with the single-cone method. Each root was sectioned horizontally to obtain three slices at 2, 5, and 10 mm from the apex. The width of the gaps at the sealer‒dentin interface from each section’s mesial and distal sides was measured under a field emission scanning electron microscope (FESEM) at×1.0 using the Digimizer software program. One-way ANOVA and post hoc Tukey tests for multiple comparisons were used to interpret and analyze the collected data.

**Results.:**

The mean gap width at the sealer‒dentin interface of C_3_ S-BG-P and Nishika Canal Sealer BG was significantly less than that of BioRoot^TM^ RCS at all root sections (*P*≤0.05). However, the mean gap width at the sealer‒dentin interface of C_3_ S-BG-P was not significantly different from Nishika Canal Sealer BG (*P*>0.05). Moreover, there were greater interfacial gaps at the apical level than at the coronal level for all the tested sealers.

**Conclusion.:**

C_3_ S-BG-P exhibited interfacial adaptation that was nearly comparable to Nishika Canal Sealer BG and superior to BioRoot^TM^ RCS.

## Introduction

 A critical factor in determining the long-term success of endodontic treatment is the complete sealing of the root canal system after the end of the chemomechanical preparation of root canals.^1^ Gutta-percha is used with a root canal sealer when obturating a root canal to increase the uniformity of root canal fillings since gutta-percha does not adhere to the root canal walls.^2^ Therefore, the root canal sealer should have desirable properties to fill any irregularities and improve the root canal filling material’s adaptability to root canal walls; otherwise, leakage and failure are more likely to occur. A properly sealed root canal system protects the root and periapical tissues from colonization and reinfection by oral microbes.^3^

 Root canal sealing product Nishika Canal Sealer BG (Nippon Shika Yakuhin Co., Ltd, Japan) is made of bioactive glass and is well known for its exceptional biological and physical properties.^4-6^ Due to the novelty of the material, studies on the sealing capacity of this sealer are relatively limited and still in the early stages. However, a calcium silicate-based sealer called BioRoot^TM^ RCS (Septodont, Saint-Maur-des-Fosses, Cedex, France) has shown several beneficial biological characteristics, such as bioactivity, biocompatibility, and antimicrobial effectiveness. On the other hand, its high solubility is considered its least desirable physical characteristic.^7,8^

 Recently, some researchers have attempted to improve the qualities of calcium silicate-based sealers by incorporating bioactive glass into them.^9-11^ With a major composition of 58% silicon dioxide, 33% calcium oxide, and 9% phosphorus pentoxide, 58s BG is one of the variable bioactive glasses. Because of its high surface area and increased porosity, it has excellent physicochemical and biological properties.^12-14^ On the other hand, exposure of calcium silicate-based materials to a phosphate-buffered saline (PBS) solution improved their characteristics, according to prior findings,^15^ but no study has included PBS in root canal sealer. In fact, root canal sealers’ physicochemical and sealing qualities could be improved by synthesizing them at the nanoscale level with a high surface area-to-volume ratio.^16^

 Several studies have used numerous sealing techniques to assess the adaptability of the filling material to root canal walls, including radioisotope tracing, electrical approaches, fluid filtering, dye penetration, a confocal laser scanning microscope, and field emission scanning electron microscopy (FESEM).^17-20^ FESEM is one of several methods used to evaluate the marginal gaps and adaptation at the sealer‒dentin interface by various studies,^17,19,20^ as the defects may be identified at the submicron level at the necessary magnification.

 Hence, this study was conducted and aimed to evaluate the width of the marginal gaps at the sealer‒dentin interface of a newly prepared nano-sealer based on tricalcium silicate and 58S bioactive glass as a powder and PBS and polyethylene glycol 400 as a liquid using FESEM at different section levels and sides from the apex of the root and compare it with two bioactive brand sealers, namely, Nishika Canal Sealer BG and BioRoot^TM^ RCS.

## Methods

###  Sealers

 This study compared a newly developed sealer (C_3_S-BG-P) with two commercially available root canal sealers, BioRoot^TM^ RCS and Nishika Canal Sealer BG. A pilot study was carried out to ascertain the percentages of the components, the powder/liquid ratio, and the duration of the mixing process utilized to create C_3_S-BG-P. The components for the C3S-BG-P and the other two sealers are listed in Table 1.

**Table 1:** Components of the tested sealers

**Sealers**	**Components**	**Manufacturer**	**Powder:liquid Proportion**
C_3_S-BG-P^*^	Powder: 45% C_3_S (80-100 nm), 30% BG ( > 60 nm), and 25% ZrO_2 _(50 nm) Liquid: 80% PBS and 20% PE	Nanochemazone_, _Edmonton, Canada PBS (Avonchem Ltd, UK) PE (Central Drug House (P) Ltd., New Delhi, India)	1 g: 0.6 mL (mixing time 1 min)
Nishika Canal Sealer BG	Paste A: Bismuth subcarbonate, fatty acid, and SiO_2_. Paste B: Calcium silicate glass, magnesium oxide, purified water, and SiO_2_	Nippon Shika Yakuhin Co., Ltd, Japan	1:1 Paste A: Paste B
BioRoot^TM^ RCS	Powder: C_3_S, ZrO_2_, and Povidone. Liquid: Aqueous solution of calcium chloride and Polycarboxylate	Septodont, Saint-Maur-des-Fosses, Cedex, France	1 scoop: 5 drops

*Experimental sealer. C_3_S = tricalcium silicate; BG = 58s bioactive glass; ZrO_2 _= zirconium dioxide; PBS = phosphate-buffered saline; PE = polyethylene glycol 400.

###  Samples collection and selection 

 Thirty human mandibular premolars were freshly extracted for orthodontic purposes. The selected teeth had straight, fully formed root apices with patent foramina, a single root and canal, and no carious lesions (root or coronal). The extracted teeth were radiographed from buccolingual and mesiodistal directions to exclude any teeth with calcifications, resorptive defects (external and/or internal), endodontic treatment, and multiple root canals. Moreover, a stereomicroscope ( × 40) (Optika, Italy) was used to exclude any tooth with cracks or defects in the root. All the teeth were placed in 250 mL of 0.1% thymol solution (BDH, England) in a closely fitted container and kept at room temperature (25 ± 2 ^o^C) until their use. The selected teeth were then cleaned with an ultrasonic scaler to remove calculus, plaque, and any remnants of organic debris on the external surface of the teeth. Afterward, they were submerged in 2.5% sodium hypochlorite (NaOCl) (Dia-Dent, Korea) for 1 hour for disinfection and then washed with distilled water for 1 hour.

###  Sample preparation 

 The selected teeth were decoronated to the cementoenamel junction (CEJ) using a diamond disc (0.2 mm thickness) (NTI^®^ Diamond Discs, Kerr, Italy) with a contra-angle handpiece (W&H, Austria) along with copious water coolant to obtain 15 mm of the root length that was confirmed with a digital caliper (SHAHE^®^, China). Moreover, any root not within 15 mm of the CEJ was discarded and replaced by another one. The root canals were accessed, and a barbed broach was used to extirpate pulpal tissues. The precise apical patency of the root canal was verified with a #10 K-file (MANI, Inc., Japan). Then, a #10 K-file was reinserted in the root canal until it became visible from the apical opening, and the working length was established at 1 mm shorter than the apical opening. Afterward, to facilitate root handling during the preparation and obturation of the samples, all the roots were vertically embedded at the center of a plastic tube (2 cm in length and 10 mm in diameter) filled with a silicon impression material with the aid of a dental surveyor. Each sample was fixed with a vice bench, and the root canal was prepared with ProTaper NEXT rotary files (Dentsply Sirona, Brazil) connected to a torque-controlled endodontic motor (Eighteenth, China) with a torque of 2 N/cm and a speed of 300 rpm according to the manufacturer’s instructions. Initially, a #15 K-file was used to construct a reproducible, smooth glide path for all the root canals. Afterward, the root canal was prepared from X1-X3 (X1 (17/.04), X2 (25/.06), and X3 (30/.07)) in the crown-down method using three up-and-down motions, and two gentle brush strokes were applied to the buccal and lingual canal walls. The irrigation regimen after each alternating file was 2.5% NaOCl (2 mL for 1 minute), normal saline (2 mL for 1 minute), 15% ethylenediamine tetra-acetic acid solution (EDTA) (2 mL for 1 minute) (Dia-Dent, Korea), and then 2 mL of normal saline was used for 1 minute. The final rinse of the canals followed the same previous regimen, except that 5 mL of normal saline for 2 minutes was used as a final rinse.The irrigant solutions were delivered using a 5-mL disposable syringe with a 30-gauge side-vented disposable needle that was entered passively 2 mm shorter than the working length, and the root canals were finally dried with X3 absorbent paper points (Dentsply Sirona, Brazil).

###  Sample assignments and obturation

 The prepared root canals were assigned randomly into three groups, with ten samples for each based on the sealer used for root canal filling. The root canal obturation was performed with a single-cone technique using a combination of gutta-percha point and sealer as follows: C_3_S-BG-P, Nishika, and BioRoot with X3 ProTaper gutta-percha cone (Dentsply Sirona, Brazil)

 Firstly, an X3 ProTaper master cone was placed within each canal to achieve tug-back to the predetermined working length. The sealer was mixed and inserted into the canal using a #30 Lentulo spiral calibrated at 300 rpm, 1 mm shorter than the working length. Sealer application continued until leakage was noted from the apical opening to ensure complete coating of the entire inner wall of the root canal with the sealer. Moreover, the master cone was dipped with a thin layer of sealer and inserted with a slow up-and-down motion inside the root canal until it reached the required working length. After that, the gutta-percha cone was seared off with a hot instrument at the level of the canal orifice, and the remaining gutta-percha was then vertically compacted with a plugger at the canal orifice. Finally, the coronal access of all the roots was sealed off with composite restoration.

 When the root canal filling was completed, the roots were removed from the plastic tubes, and radiographs were taken from the buccal and mesial directions to check the quality of the root canal filling. Afterward, the roots were wrapped in moist paper and incubated at 95% humidity at 37 ^o^C for one week to ensure the complete setting of the sealer and simulate the clinical situation.

###  Analysis of interfacial adaptation

 The first 3 mm from the coronal portion of each root was embedded vertically with the aid of a surveyor into a cylindrical acrylic resin mold (10 mm in diameter and 15 mm in length). After that, each mold was fixed to a special design standardized table with a bench vice, and the root was sectioned perpendicular to its long axis with a diamond disc (0.2 mm in thickness) in a slow-speed straight handpiece under constant water coolant. The roots were sectioned at 2 mm, 5 mm, and 10 mm from the apex, and three 1-mm-thick slices were achieved (checked with a digital caliper). The apical surface of each slice was polished for 10 seconds with abrasive sandpaper. Then, each section was submerged in a 15% EDTA solution for 30 seconds, then in 2.5% NaOCl for 30 seconds, and finally rinsed with distilled water to remove the smear layer. The specimens were placed in a dissector for dehydration at 37^o^C for 24 hours. Sealer‒dentin interface adaptation was evaluated by FESEM (TESCAN MIRA3, France) for the apical face of each slice at mesial and distal sites. The apical surface of the slice was sputtered and coated with a thin film of gold (20 nm). Then, each slice was mounted in carbon adhesive stubs, which were then viewed under FESEM at 15 kV accelerating voltage, 10 mA, and a magnification of × 1000. The obtained micrographs for each sample were imported to the Digimizer software program to measure the gaps (μm) between the sealer and dentin at different positions in each section’s mesial and distal sites using a calibrated measuring tool. After that, the measure readings were averaged to achieve a single mean value for each section and recorded.

###  Statistical analysis

 After confirming the data’s normality with the Kolmogorov-Smirnov test, the data were analyzed and interpreted using one-way ANOVA and Tukey tests for multiple comparisons in the Statistical Package for the Social Sciences (SPSS) software, version 26 (IBM, Armonk, New York). The significance level was defined at *P* ≤ 0.05.

## Results

**Figure 1:**
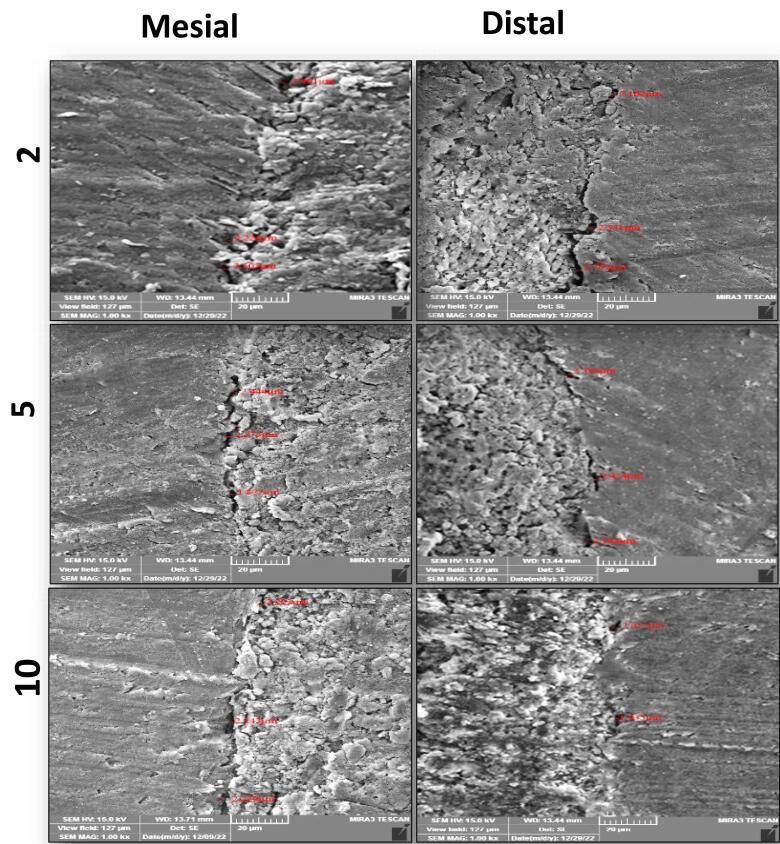
Representive FESEM images ( × 1000) of the interfacial adaptation of C3S-BG-P to radicular dentin at different sections from the apex

**Figure 2:**
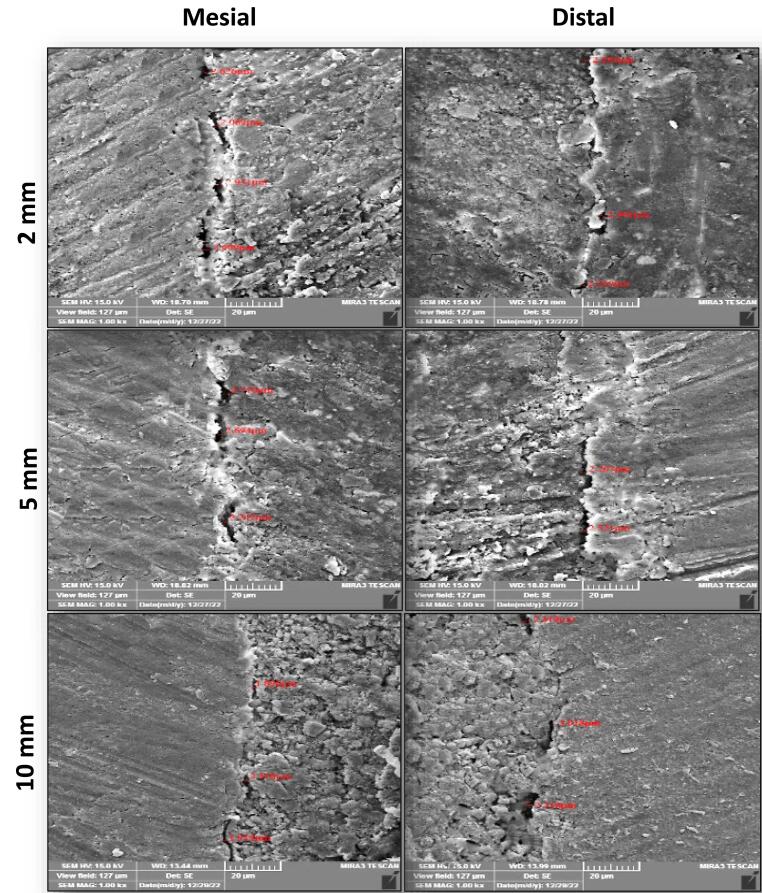
Representive FESEM images ( × 1000) of the interfacial adaptation of Nishika to radicular dentin at different sections from the apex

**Figure 3:**
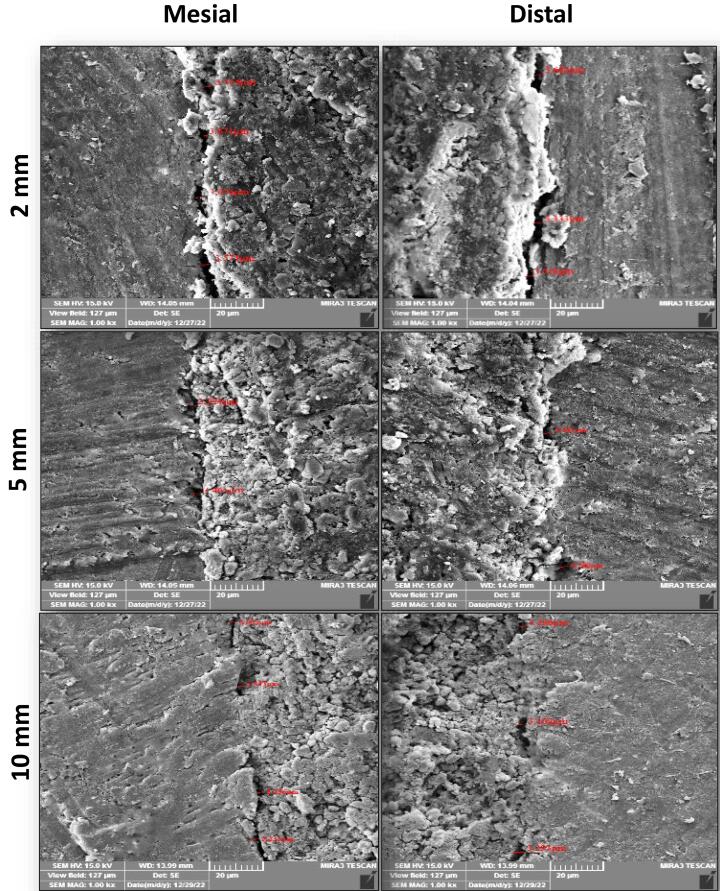
Representive FESEM images ( × 1000) of the interfacial adaptation of BioRoot to radicular dentin at different sections from the apex

 FESEM images of the interfacial adaptation of the tested sealers to radicular dentin at different root sections are presented in Figures 1–3.

 Comparing the mean gap width at the sealer‒dentin interface between the tested sealers at each root section from the apex (2 mm, 5 mm, and 10 mm), ANOVA revealed a significant difference between the tested sealers at all root sections. Then, a Tukey test was performed to show exact differences (Table 2).

**Table 2:** Comparisons of the interfacial adaptation of the tested sealers to the radicular dentin at different sections from the apex

**Root sections**	**Tested sealers**	**Mean (µm)±SD**	**F-value**	***P*** value^*^
2 mm	C_3_S-BG-P	2.43 ± 0.036^a^	5018.62	0.000
	Nishika	2.46 ± 0.021^a^		
	BioRoot	3.55 ± 0.025^b^		
5 mm	C_3_S-BG-P	2.40 ± 0.049^a^	2270.51	0.000
	Nishika	2.44 ± 0.019^a^		
	BioRoot	3.39 ± 0.036^b^		
10 mm	C_3_S-BG-P	2.36 ± 0.102^a^	341.168	0.000
	Nishika	2.40 ± 0.105^a^		
	BioRoot	3.35 ± 0.078^b^		

SD,Standard deviation; C_3_S-BG-P, Experimental sealer. Note:The variable letters vertically mean significant differences exist. Comparison was done alone for each root section.^*^P ≤ 0.05 means significant difference.

 The Tukey test showed that the mean gap width of C_3_S-BG-P and Nishika was significantly less than that of BioRoot at all root sections. However, the mean gap width of C_3_S-BG-P was less than that of Nishika but was not significantly different.

**Figure 4:**
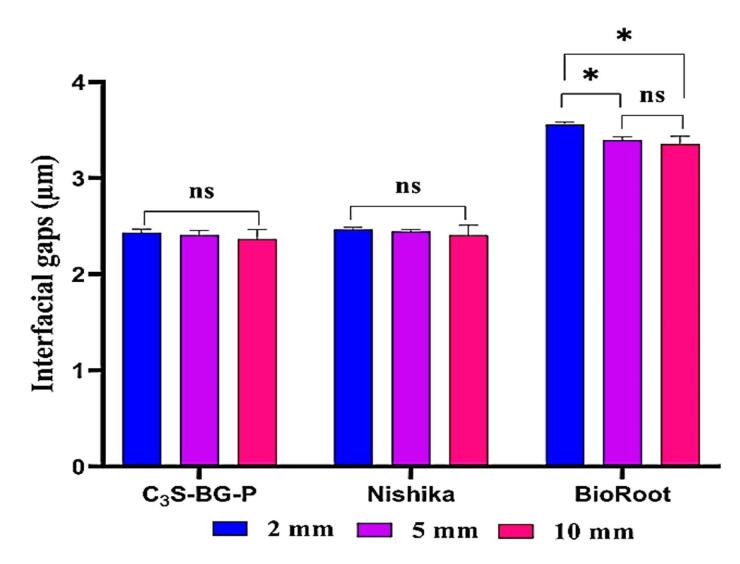
Bar charts illustrate the interfacial gaps of the tested sealers to the radicular dentin at different sections from the apex. ns means no significant difference. * Indicates a significant difference at *P* ≤ 0.05

 A comparison of the results of each tested sealer at each root section showed that the mean gap width increased from coronal to apical sections for all the tested sealers. However, there were no significant differences between C_3_S-BG-P and Nishika in all the sections. However, there was a significant difference in mean gap width between 2-mm sections and 5-mm and 10-mm sections for BioRoot. However, the 5-mm and 10-mm sections showed no significant differences in the mean gap widths for the BioRoot to radicular dentin (Figure 4).

## Discussion

 The root canal treatment’s success depends on three-dimensional obturation and sealing of the root canal systems, which prevents apical and coronal ingress of fluids and microbes and the subsequent reinfection, promotes healing of periapical lesions, and strengthens the root dentin.^21^ Therefore, the root canal sealers should have adequate adaptation and adherence properties to fill voids, accessory and lateral canals, and imperfections in the root canals, as well as packing any minor discrepancies between the root canal dentin and core materials.^18,20^

 In the current study, the specimens were cross-sectioned horizontally, and the marginal gaps between the root canal wall and filling material were evaluated using FESEM. This made it possible to monitor the adaptation and estimate flaws throughout the entire canal lumen at the selected sides and levels for a more accurate and thorough assessment.^19^ Therefore, using the FESEM allowed estimation of the mean gap width at the sealer‒dentin interface for all tested sealers at chosen root sections from the apex (2 mm, 5 mm, and 10 mm) on the mesial and distal sides.

 Based on the findings of the present study, the mean gap width of C_3_S-BG-P and Nishika was significantly less than that of BioRoot at all root sections. However, the mean gap width of C_3_S-BG-P was less than that of Nishika but was not significantly different.

 The sealer adaptation depends on many factors, including smear layer removal, root canal dimension, dentin permeability, dentinal tubule diameter, physical and chemical properties, constituents, particle size, consistency, surface tension, and contact angle of the root canal sealers.^21-23^

 The higher adaptation of the C_3_S-BG-P and Nishika than that of BioRoot resulted mainly from small particle size, high flowability, low film thickness, low solubility, and better consistency,^24^ which enhanced the penetration of the sealer into micro-irregularities, accessory canals, isthmus, and dentinal tubules. Arikatla et al^2^ and Pius et al^25^ reported that the high flow rate and low solubility of the AH Plus sealer resulted in greater penetration depth and better marginal adaptation compared to the BioRoot. The high solubility of the BioRoot resulted in more voids being formed within the materials, which might affect the adaptation of the sealer to the canal walls.^26^

 Moreover, the high viscosityof the BioRoot, due to its contents of povidone and polycarboxylate,^27,28^ resulted in the lowest interfacial adaptation than that of the C_3_S-BG-P and Nishika. Also, Desouky et al^29^ verified that nano-sized sealers effectively prevent leakage during root canal treatment. Similarly, other findings have confirmed that using small particles decreases the contact angle and increases the surface area of the sealer, thereby enhancing the spread and adaptation of the sealer into lateral and accessory root canals.^22,23^ Therefore, the small particle size and greater fluidity of the C_3_S-BG-P and Nishika allowed their better adaptation to root canal walls. Chew et al^30^ compared three different sealers (EndoSeal MTA, CeraSeal, and Nishika Canal Sealer BG) with an AH Plus in oval root canals. Through the evaluation of the interfacial adaptability and depth of penetration at 3, 6, and 9 mm root sections from the apex under a confocal laser scanning microscope, they concluded that Nishika resulted in superior penetration and adaptation than the other tested sealers due to its superior physicochemical properties and fine particle size.

 Moreover, the C_3_S-BG-P exhibited better adaptation because it contained PBS and bioactive glass (BG), which improved the chemical absorption of silicon and calcium by dentin. This led to the formation of a mineral infiltration zone, which improved the penetration of sealer minerals (calcium, carbon, and silica) into inter-tubular dentin after collagen fibers were denatured by the alkaline effects of the sealer’s byproducts.^31^ This is consistent with other findings that reported that the PBS within the root canal enhanced the adaptation and bond strength of calcium silicate-based sealers to radicular dentin.^32,33^ Also, Marissa et al^22^ reported that calcium silicate sealers that contained phosphate exhibited better dentinal tubule penetration than those with pure calcium silicate. Another finding by Waly and Salama^34^ showed that incorporating the BG in the root canal sealers improves their bond strength to radicular dentin.

 Nevertheless, the main hydration byproduct of C_3_S-BG-P is hydroxyapatite, which promotes remineralization, interfacial adaptation, and sealer infiltration in the tubules, thereby reducing marginal gaps at the interface and increasing the bond strength of the filling to the dentin and the tooth’s resistance to fracture.^35,36^

 Regarding all of the sealers in this investigation, there were greater interfacial gaps at the apical than the coronal level. The lower diameter and density of dentinal tubules present at the apical area have been suggested as the causes of this difference. Additionally, the apical third has more sclerotic dentin devoid of tubules and morphological diversity than the coronal third.^19,20^ Another explanation could be that it is difficult to extend the endodontic tools to the apical third, which makes it difficult to deliver enough irrigants; compared to the coronal third of the canal, this can result in less removal of the smear layer.^37^

 The current finding is limited by the fact that the adaptation of the tested sealers was evaluated in vitro. This type of assessment does not replicate an in vivo or clinical situation. Therefore, further research on root canal filling in animal models should be performed to investigate its impact on potential clinical applications.

## Conclusion

 According to the findings of the present investigation, the newly prepared C_3_S-BG-P nano-sealer has an interfacial adaptation that is nearly comparable to Nishika Canal sealer BG and superior to that of BioRoot^TM^ RCS. Further studies are necessary to determine the correlation between sealer penetration into the dentinal tubules and interfacial adaptation or marginal gaps, as well as to evaluate the adaptability and penetration of the sealer with a method other than FESEM.

## Acknowledgments

 We would like to sincerely thank the College of Dentistry, the University of Mosul for all of its assistance.

## Competing Interests

 None.

## Ethical Approval

 This study was carried out in accordance with all the standards and policies of the local human subjects oversight committee, and it was approved by the College of Dentistry, the University of Mosul, Mosul, Iraq, by the Research Ethics Committee (REC). The study’s REC reference number is UoM.Dent/A.H.71/22 on November 21, 2022.

## Funding

 None.
